# Editorial: Cellular Therapies in Cancer

**DOI:** 10.3389/fimmu.2019.02788

**Published:** 2019-11-27

**Authors:** Hind Rafei, Rohtesh S. Mehta, Katayoun Rezvani

**Affiliations:** ^1^Division of Cancer Medicine, MD Anderson Cancer Center, Houston, TX, United States; ^2^Department of Stem Cell Transplant and Cellular Therapy, MD Anderson Cancer Center, Houston, TX, United States

**Keywords:** cellular therapy, chimeric antigen receptor-T cell therapy, chimeric antigen receptor-natural killer cell therapy, adoptive cell therapy, T cells, natural killer cells, immune effector cells, cancer

The field of cellular therapy is evolving rapidly with novel therapeutic modalities that include a wide spectrum of products. These include tumor-infiltrating lymphocytes (TILs), engineered T-cell receptor (TCR), chimeric antigen receptor (CAR)-T cells, cytotoxic T lymphocytes (CTLs), natural killer (NK) cells, and others. Among these, CAR T-cell therapy was the first to be approved by the US Food and Drug Administration (FDA) for the treatment of patients with B lymphoid malignancies. Specifically, Tisagenlecleucel (Kymriah) was approved in August 2017 for the treatment of patients with refractory B-cell acute lymphoblastic leukemia (ALL) up to the age of 26, and in May 2018, for adults with refractory B-cell lymphoma and Axicabtagene Ciloleucel (Yescarta) was approved for adults with refractory B-cell lymphoma in October 2017. However, a number of challenges have hindered the widespread use of engineered immune cells beyond B-cell malignancies. Some of these challenges include the lack of ideal targets for solid tumors, antigen loss mediating cancer relapse, the complexity of generating a patient-specific cell product, toxicity owing to the “on-target off-tumor” reactivity and cytokine release syndrome/neurotoxicity, inefficient trafficking to tumor sites, and tumor-mediated immunosuppression, to name a few. Advances in synthetic biology and genetic engineering, and the investigation of new platforms for cellular therapies have paved the road for the development of strategies to bypass some of these challenges, in order to advance the field forward and make a wider clinical impact. This collection of published articles is comprised of a series of original research and reviews highlighting recent advances in different forms of cellular therapies employing both T and NK cells against hematologic malignancies as well as solid tumors.

∎ Hoffmann et al. addressed a major question regarding the phenotype of CAR-T cells generated from untreated chronic lymphocytic leukemia (CLL) patients and specifically the ratio of naïve to effector T cells (T_N_/T_E_) in the final product and their expansion and persistence following adoptive transfer. The authors found that compared to healthy donors, the T_N_/T_E_ ratio was low in CAR-T products generated from untreated CLL patients, constituting a challenge for long-lasting CAR-T effects. As the high proportion of malignant B cells in CLL patients may hinder expansion of CAR-T_N_ cells, the authors suggested that depletion of CLL cells prior to CAR-T production may improve the quality of the product (Hoffmann et al.).
∎ Qin et al. also addressed the survival and functionality of the transferred T cells, but from another angle through exploring different culture conditions to generate more effective CTLs for adoptive therapy. They proposed a new protocol to generate potent CTL clones using mouse embryonic fibroblast-conditioned medium (MEF-CM) and showed that these cells have higher potential to persist long-term in tumor-bearing and non-tumor bearing mice. Characterization of these cells suggests that MEF-CM enhances the effector functions of CD8^+^ T cells and thus augmenting antitumor immunity (Qin et al.).
∎ Khan et al. investigated another important problem concerning the paucity of tumor antigens in the development of cellular therapies. The authors report overexpression of cathepsin G (CG) in cell lines and in primary ALL blasts and investigated CG as a tumor target for immunotherapy of ALL. Specifically, they showed that CG1-specific CTLs efficiently kill primary ALL blasts *in vitro* (Khan et al.).
∎ Houghtelin and Bollard reviewed the role of virus-specific T cells (VSTs) as a minimally toxic therapy in immunocompromised patients with viral infections. They also discuss future directions, including the use of naïve donor sources and third-party banks (Houghtelin and Bollard).
∎ Parisi et al. reviewed the role of alloreactive NK cells against acute myeloid leukemia (AML), and assessed biomarkers predictive of response, dosing, and other aspects which could maximize their efficacy.
∎ Herrera et al. explored a method to generate a large number of mature NK cells that could be used in NK cell-based immunotherapy. Using OP9 feeder cells, they were able to generate large numbers of mature and functional NK cells from umbilical cord blood (Herrera et al.).
∎ Lieberman et al. similarly aimed to explore an optimal approach to expand NK cells. They used a clinically validated expansion protocol using an engineered K562 cell line expressing membrane-bound interleukin (IL)-15 and 4-1BB ligand (K562-mbIL-15-4-1BBL) as feeder cells together with exogenous high dose IL-2 in the culture medium. They showed that NK cells expanded *ex vivo* in the presence of K562-mbIL-15-4-1BBL are not terminally mature but instead have high cytokine production capacity and antitumor efficacy. They concluded that this method could be an ideal source of long-lived and highly potent NK cells for cellular therapy (Lieberman et al.).
∎ Mehta and Rezvani reviewed the approach of genetically engineering NK cells to express a CAR (CAR-NK cells). As NK cells do not cause graft-vs.-host disease (GVHD), they can be used as a readily available “off-the-shelf” cellular therapy that could increase accessibility of this treatment for many more patients (Mehta and Rezvani).

Despite impressive successes in the management of CD19+ B-cell hematologic malignancies, progress in the solid tumor field has been met with a number of challenges. This is due to several factors, such as limited trafficking of adoptively transferred immune cells into the solid tumor sites, and the immunosuppressive tumor microenvironment. Importantly, identifying specific tumor antigens that are highly and uniformly expressed among solid tumors with low expression on normal tissues has been challenging. Adoptive transfer of TILs as an anti-cancer therapy pioneered by Steven Rosenberg at the National Institutes of Health (NIH) over two decades (1) has resulted in significant objective response rates in patients with metastatic melanoma and is being explored in other cancer settings (2). TCR-T cell therapy has also been commonly explored in solid tumors and is showing promise in some clinical trials (3). In addition, growing efforts are being made to expand the use of CAR-based therapies for solid tumors.

∎ Seliktar-Ofir et al. investigated a method to select tumor-specific TILs instead of the classic randomly isolated TILs. As CD137 is a co-stimulatory marker induced upon the interaction of T cells with their target cells, they explored a method to select CD137-expressing T cells *ex vivo*. CD137-selected TILs had superior antitumor activity and were enriched for T cells recognizing neoantigens and shared tumor antigens (Seliktar-Ofir et al.).
∎ Lo Presti et al. reviewed the use of a minor, yet clinically significant, population of T lymphocytes- the γδ T-cells. These cells have distinctive features justifying their use in anti-cancer immunotherapy; namely, they are not subject to major histocompatibility complex restriction, they are less dependent on co-stimulatory molecules than αβ T-cells, and they have known cytotoxicity against cancer cells (Lo Presti et al.).
∎ Jiang et al. presented their research in CAR T-cell therapy for hepatocellular carcinoma (HCC). They established patient-derived xenografts (PDX) mouse models of HCC and evaluated the cytotoxicity of anti-glypican 3 (GPC3)-CAR-T cells *in vivo*. GPC3 CAR-T cells efficiently suppressed tumor growth in one model, and eradicated the tumor in two other models where GPC3 was highly expressed (Jiang et al.).
∎ Mirzaei et al. reviewed the advances made in CAR T-cell therapy for solid tumors. They outlined the barriers to the effectiveness of this approach in solid tumors and some of the strategies to overcome these (Mirzaei et al.).
∎ Le and Thai reviewed the application of T- and NK-cell based immunotherapies in neuroblastoma and other childhood solid tumors such as other central nervous system tumors and sarcomas. They also delineated key differences between adult and fetal/neonatal immune systems (Le and Thai).
∎ Uppendahl et al. focused on the role of NK cell-based immunotherapy in gynecologic malignancies. They reviewed the advances made in harnessing these cells for clinical application against ovarian, cervical and uterine cancer (Uppendahl et al.).
∎ Tian et al. sought to enhance the activity of NK cells against HER2^+^ breast cancer. Based on previous research showing that Herceptin could increase the cytotoxicity of lymphocytes against HER2 expressing tumors (4), they investigated the effect of engaging NK cells with Herceptin on their antitumor activity. They found that Herceptin increased NK cell proliferation, migration and cytotoxicity against HER2^+^ breast cancer cells. In a pilot study, Herceptin-treated NK cells were administered to a HER2^+^ breast cancer patient who could not tolerate the cardiotoxic side effects of Herceptin, leading to shrinkage in lung nodular metastases (Tian et al.).

In summary, we anticipate that this collection of original articles and reviews will increase our understanding of the progress made in the field of cellular immunotherapies for cancer and will serve as an inspiration for future research efforts. Despite the numerous challenges encountered in moving the field of cellular therapy forward, the success achieved thus far is exciting and promising for a paradigm shift in the years to come. With the exploding advances in genome editing and synthetic biology, efforts from around the world will continue to advance the field toward curative approaches for highly resistant cancers.

## Author Contributions

All authors listed have made a substantial, direct and intellectual contribution to the work, and approved it for publication.

### Conflict of Interest

MD Anderson Cancer Center and Takeda have entered a license agreement and research agreement to develop CB-CAR NK cells for the treatment of B-cell malignancies and other cancers, which creates an institutional conflict of interest under MD Anderson policy. KR has received an education grant from Affirmed and Pharmacyclics; is a consultant for EMD serono and Virogin; and is a member of the Kiadis data and safety monitoring board. The remaining authors declare that the research was conducted in the absence of any commercial or financial relationships that could be construed as a potential conflict of interest.
